# Case Report: Could genetic factors influence the outcomes of first-line enfortumab vedotin plus pembrolizumab therapy in patients with metastatic urothelial carcinoma? Two cases of patients harbouring a BRCA mutation

**DOI:** 10.3389/fonc.2025.1648230

**Published:** 2025-10-03

**Authors:** Giuseppe Salfi, Chiara Maria Agrippina Clerici, Giovanna Pecoraro, Martino Pedrani, Marialuisa Puglisi, Luigi Tortola, Ricardo Pereira Mestre, Ursula Vogl, Ilaria Colombo, Jessica Barizzi, Milo Frattini, Rossella Graffeo, Stefania Rizzo, Andrea Gallina, Fabio Monni, Nicola Fossati, Silke Gillessen, Fabio Turco

**Affiliations:** ^1^ Oncology Institute of Southern Switzerland (IOSI), Ente Ospedaliero Cantonale (EOC), Bellinzona, Switzerland; ^2^ Institute of Oncology Research (IOR), Bellinzona, Switzerland; ^3^ Department of Clinical Medicine and Surgery, University of Naples “Federico II”, Naples, Italy; ^4^ School of Specialization in Medical Oncology, Department of Human Pathology “G. Barresi”, University of Messina, Messina, Italy; ^5^ Faculty of Biomedical Sciences, Università della Svizzera Italiana, Lugano, Switzerland; ^6^ Istituto Cantonale di Patologia, Ente Ospedaliero Cantonale (EOC), Locarno, Switzerland; ^7^ Imaging Institute of Southern Switzerland, Ente Ospedaliero Cantonale, Lugano, Switzerland; ^8^ Urology Service, Department of Surgery, Ente Ospedaliero Cantonale, Università della Svizzera Italiana, Lugano, Switzerland

**Keywords:** metastatic urothelial carcinoma, enfortumab vedotin, pembrolizumab, EV-302, BRCA, genomic biomarkers

## Abstract

The introduction of enfortumab vedotin combined with pembrolizumab (EV-P) as a first-line treatment for advanced urothelial carcinoma (UC) has transformed the therapeutic landscape and holds great promise for improving patient outcomes. However, predictive and prognostic biomarkers for this novel regimen remain limited, and no specific subgroup has yet been identified for whom frontline EV-P could be withheld in favor of platinum-based chemotherapy. We report the first two cases of patients with BRCA-mutant metastatic UC who experienced markedly short progression-free survival with first-line EV-P but achieved more durable responses with second-line platinum-based chemotherapy. These observations raise important questions about the potential predictive role of BRCA - and more broadly, DNA damage repair - mutations in the evolving treatment paradigm of UC. Given the known sensitivity of BRCA-mutated tumors to platinum agents, frontline platinum-based chemotherapy may warrant consideration in this molecularly defined subgroup. Larger studies are needed to validate these preliminary findings and inform treatment selection.

## 1 Introduction

Urothelial carcinoma (UC) is the 10th most common malignancy worldwide. It is the most frequent cancer of the urinary tract, and approximately 10% of cases arise from the upper tract (UTUC) (1). Metastatic UC (mUC) is associated with an aggressive disease course and a 5-year survival rate of 5-8% (1), with metastatic UTUCs being associated with a worse prognosis (2).

The results of the III EV-302/KEYNOTE-39A trial have recently transformed the treatment landscape of mUC (3). In this study the combination of the antibody drug conjugate (ADC) enfortumab vedotin with the immune checkpoint inhibitor pembrolizumab (EV-P) as first-line treatment in patients with mUC demonstrated improved oncological outcomes compared to platinum-based chemotherapy. In particular, patients treated with EV-P had both a median progression-free survival (PFS) and a median overall survival (OS) that was almost doubled compared to patients treated with platinum-based chemotherapy (12.5 and 31.5 months vs 6.3 and 16.1 months, with an HR of 0.45 for PFS and 0.47 for OS). Patients treated with EV-P had an overall response rate (ORR) of 67.7%, with only 8.7% experiencing progressive disease as their best response.

The marked benefit in favor of EV-P was maintained regardless of the primary site of origin of disease (upper vs lower tract), PD-L1 expression (low vs high), cisplatin eligibility status (eligible vs ineligible) and site of metastasis (visceral vs lymph nodes). Based on these impressive results, international guidelines have included EV-P as the new standard of care for first-line therapy in patients with mUC who are eligible for this combination therapy (4, 5). However, predictive factors for EV-P efficacy remain largely unknown, highlighting a critical gap in knowledge.

The use of information derived from immunohistochemistry analysis or from next generation sequencing is becoming fundamental to personalize the treatment of patients with mUC (4, 5). For example, patients with mUC with FGFR alterations (FGFR2/3 mutations or FGFR3 fusions) can benefit from FGFR inhibitors, such as erdafitinib (6). In addition, there are interesting preliminary data for the use of Fam-trastuzumab deruxtecan-nxki in patients with HER-2 positive mUC as assessed by immunohistochemistry. This treatment has been included among the possible therapeutic options for HER-2 positive disease in advanced lines of therapy in the current NCCN guidelines (4) following the results of the DESTINY-PanTumor02 phase II Trial (7).

To date, we do not know whether the efficacy of EV-P as first-line therapy in patients with mUC can be influenced by specific molecular features. It is therefore possible that some subgroups of patients with certain mutations may still benefit more from a platinum-based regimen as first-line treatment than from EV-P.

DNA damage response and repair (DDR) gene mutations occur in up to 25% of UC cases, with *BRCA1* and *BRCA2* mutations present in approximately 3% and 4.5% of tumors, respectively (8).

Here, we present two cases of *BRCA*-mutant metastatic UTUC treated with first line EV-P followed by second-line platinum-based chemotherapy, who had a short response to EV-P and a longer response to second line platinum-based chemotherapy.

## 2 Case description

### 2.1 Case one

**Figure 1:**
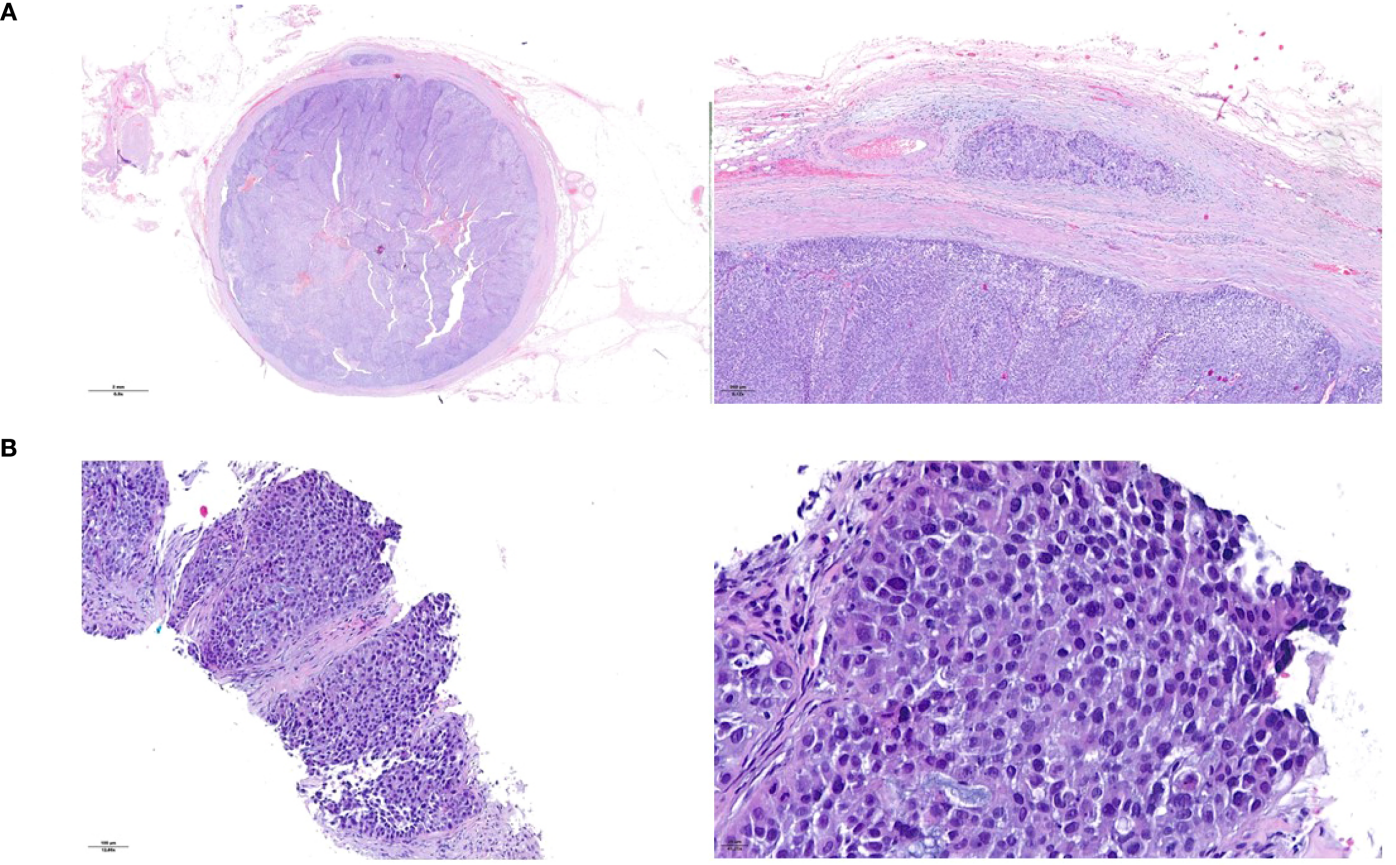
Histopathological presentation of the urothelial ureteral primary tumor **(A)** and of the peritoneal metastasis **(B)**. Hematoxylin and eosin staining.

A 75-year-old Caucasian non-smoker male presented with worsening lower urinary tract symptoms during a routine urology visit. A contrast enhanced CT scan revealed left ureteral dilation with a filling defect in the pre-vesical and intramural ureter. A left distal ureteral resection with ureteral reimplantation was performed, and pathological analysis confirmed high-grade invasive urothelial carcinoma with muscle invasion (pT2) (**Figure 1A**). Concurrent bladder resection revealed multifocal high-grade pT1 papillary UC.

Staging CT of the chest and abdomen identified solid lesions in the left renal pelvis and ureter, bladder wall thickening, ascites, and peritoneal nodules. A biopsy of a peritoneal nodule revealed poorly differentiated carcinoma of urothelial origin (**Figure 1B**). The final diagnosis was pT2N0M1, stage IV UTUC.

Somatic next generation sequencing (NGS) evaluation of the ureteral specimen was performed by Ion Torrent (Thermo Fisher Scientific, Waltham, MA) using Oncomine Comprehensive Assay v3 (Thermo Fisher Scientific, Waltham, MA) and identified a BRCA2 p.S2887* variant, a TP53 p.P151Sfs*13 variant, and increased copy numbers of CCND1, FGF19, FGF3, PPARG, and RAF1. The tumor was classified as microsatellite-stable while tumor mutational burden analysis was not performed. The case was discussed at our molecular tumor board where the BRCA alteration found was considered pathogenic and with an elevated allele frequency (69.37%). Considering the current evidence and recommendations (9), patient was referred for germinal testing, which did not show any pathogenic variants in BRCA2 or the other examined genes.

**Figure 2:**
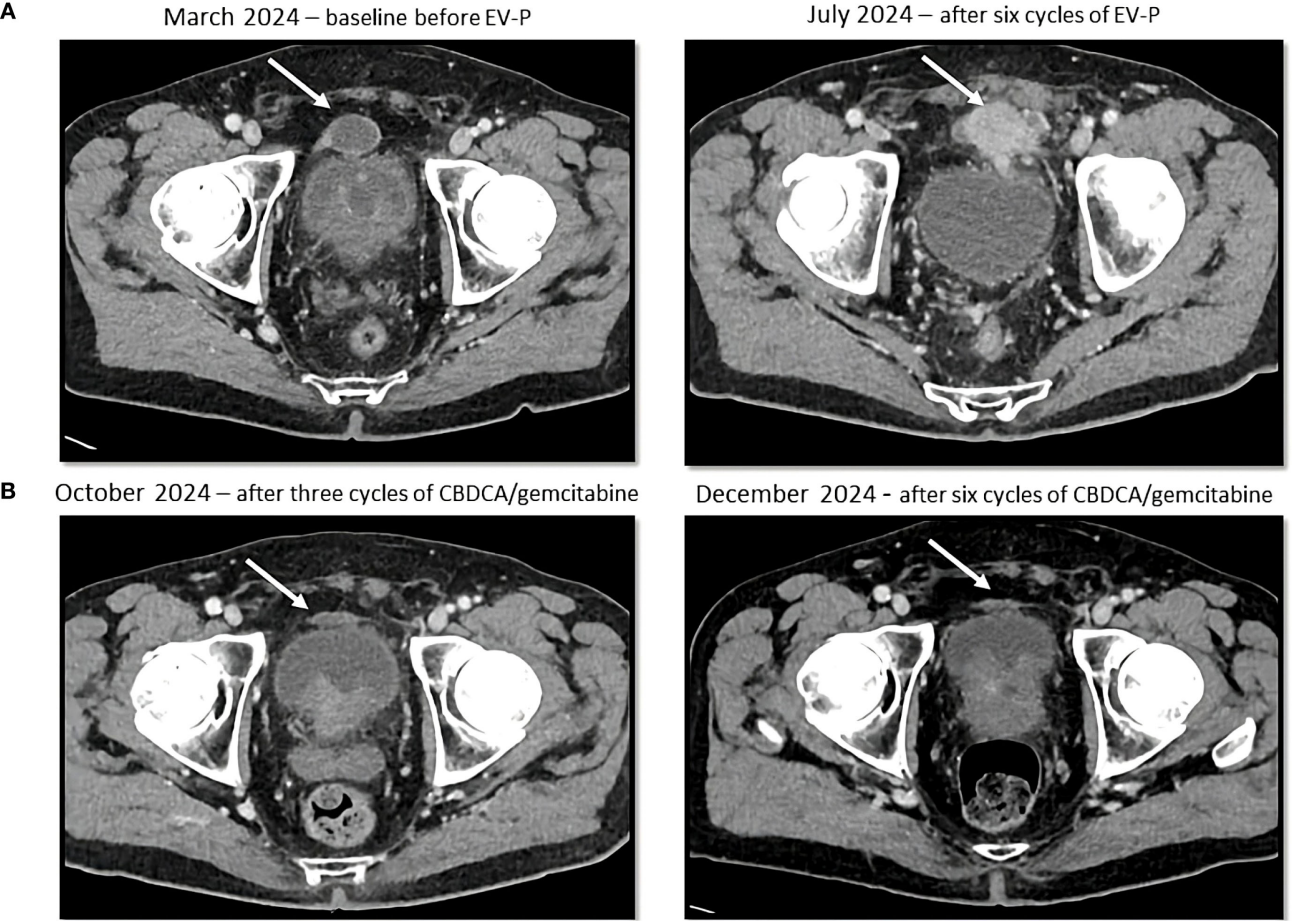
Sequential CT scans showing disease progression during treatment with enfortumab vedotin and pembrolizumab **(A)** and partial response with platinum-based chemotherapy **(B)**. Cystic lesion (white arrow) above the bladder, that increased in density and size during enfortumab vedotin and pembrolizumab treatment and then decreased in size after platinum-based therapy administration.

The patient was referred to the Oncology Department and in March 2024 started first line EV-P treatment. A CT scan was performed after the administration of the third cycle of the treatment and showed a RECIST 1.1 partial response (PR), with reduction in pyelo-ureteral lesions and peritoneal nodules, as well as a complete resolution of the ascites. However, a single pre-vesical peritoneal lesion had increased in size (8mm diameter increase, from 13 to 21 mm). A follow-up CT after three additional cycles (July 2024) demonstrated progressive disease, with new peritoneal lesions, suspected left pyeloureteral junction disease relapse, and an enlarged right obturator lymph node (**Figure 2A**). EV-P treatment was therefore discontinued after six cycles. All treatment cycles were administered without dose reduction or delay. During the treatment, the patient developed grade 1 oral mucositis and peripheral neuropathy.

Second-line carboplatin AUC 5 day 1 plus gemcitabine 1000 mg/m² day 1 and day 8 every 3 weeks was subsequently initiated in August 2024. Carboplatin was chosen because of a mildly reduced glomerular filtration rate of 50 mL/min/1.73 m^2^.

A CT scan after three cycles (October 8, 2024) showed a PR by RECIST 1.1 with a significant reduction in tumor burden with a reduction in size of numerous peritoneal lesions (the biggest being the aforementioned prevescical lesion that decreased from 46 mm to 31 mm) and of the right obturator lymphadenopathy (with a short axis from 10 mm to 6 mm). The case was subsequently discussed at our multidisciplinary board which, due to the good treatment response, suggested continuing treatment with carboplatin and gemcitabine for up to six total cycles. Subsequent cycles were administered at a reduced dose (25% dose reduction for both drugs), due to grade 2 anemia and grade 1 peripheral neuropathy. The CT scan performed after six cycles (December 18, 2024) confirmed an even deeper response, with complete response of some peritoneal nodules (**Figure 2B**). A follow-up CT scan in March 2025 further demonstrated the stability of the achieved response.

The patient remains in follow-up with no evidence of disease progression, currently eleven months post-second-line treatment initiation, and seven months from the last cycle of platinum-based chemotherapy.

### 2.2 Case two

**Figure 3:**
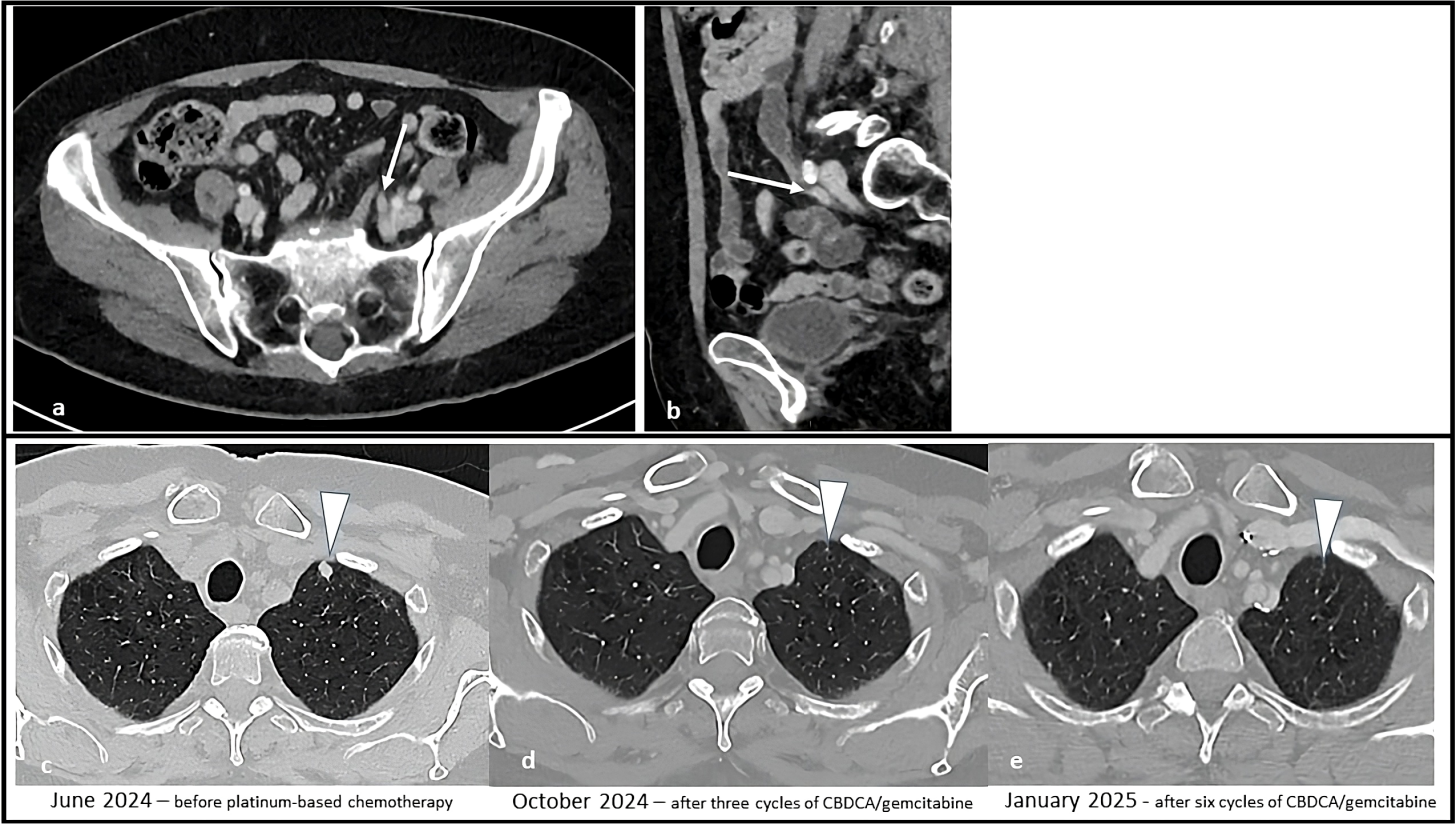
Sequential CT scans showing disease progression during treatment with enfortumab vedotin plus pembrolizumab and subsequent partial response with platinum-based chemotherapy. Solid tissue within the left ureter causing hydronephrosis (white arrow) in the axial **(a)** and sagittal **(b)** CT images. Afterwards, the appearance of solid lung nodules was noted [white arrowhead in **(c)**], that progressively responded to therapy **(d)** and disappeared **(e)**.

A 69-year-old Caucasian female smoker presented with pelvic pain and macrohematuria. A CT scan revealed a suspicious tissue mass in the distal third of the left ureter, with ureteral dilation but no other suspicious urinary tract lesions or lymphadenopathies (**Figure 3**). She subsequently underwent robot-assisted left nephroureterectomy with bladder cuff excision and pelvic and hilar loco-regional lymphadenectomy. Histological analysis confirmed high-grade urothelial carcinoma with infiltration of the muscularis propria, focal angioinvasion, and negative resection margins.

**Figure 4:**
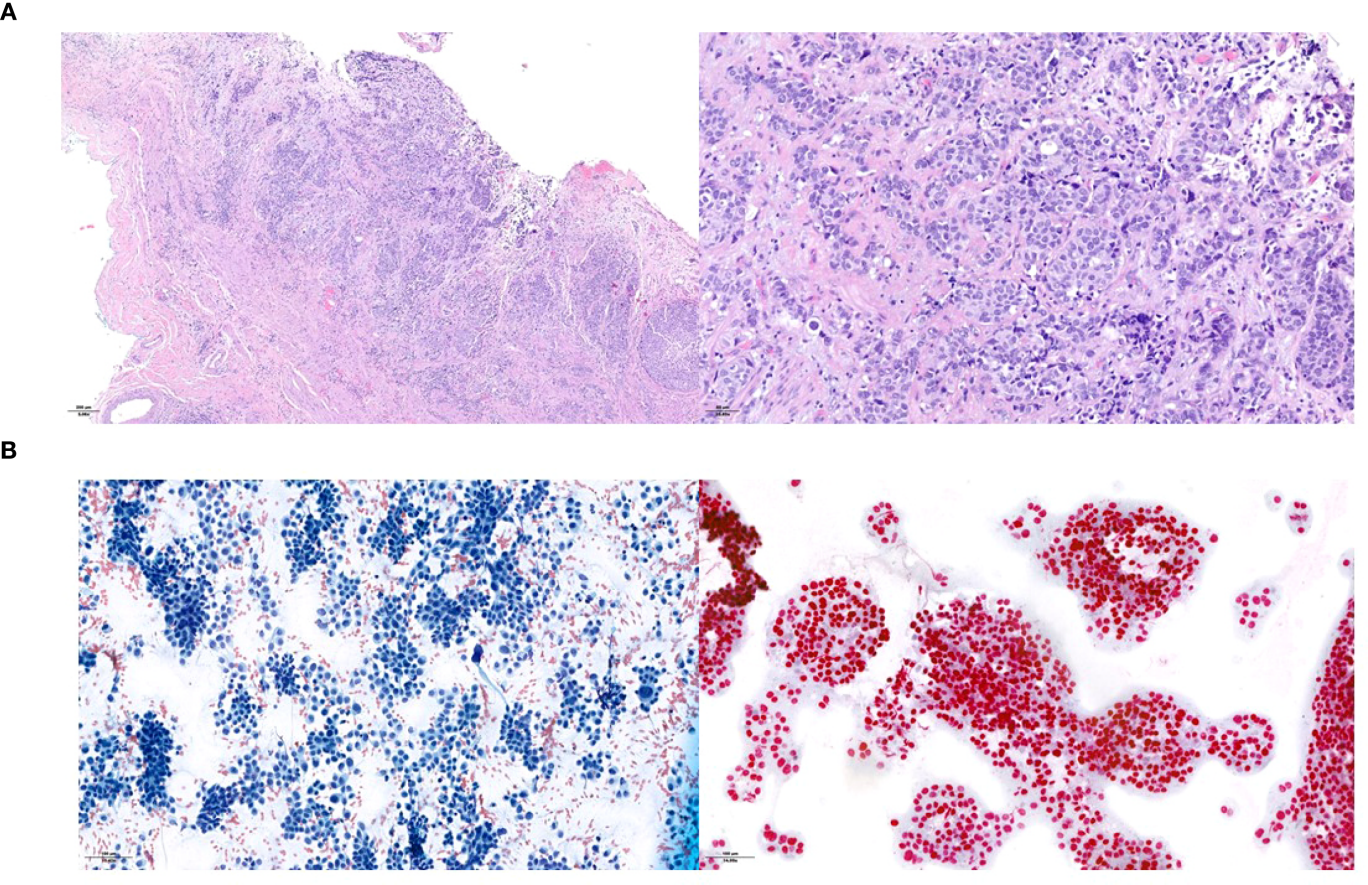
Histopathological presentation of the urothelial ureteral primary tumor **(A)** and of the supraclavicular lymph node metastasis **(B)**. Hematoxylin and eosin staining in the primary tumor and assessment of neoplastic cellularity and GATA-3 expression in the lymph node.

The final pathological staging was pT2, L/V1, Pn0, G3, R0. However, carcinomatous lymphangitis was also noted in the adipose tissue adjacent to the left common iliac lymph nodes (**Figure 4A**).

Somatic NGS evaluation of the ureteral specimen was performed by Ion Torrent (Thermo Fisher Scientific, Waltham, MA) using Oncomine Comprehensive Assay v3 (Thermo Fisher Scientific, Waltham, MA) and identified an isolated BRCA2 p.N2930Ifs*7 mutation with a 25.5% allele frequency. The case was discussed at our molecular board where the BRCA alteration found was considered pathogenic. Germline genetic testing did not reveal any pathogenic variants (specifically in BRCA2) but identified three variants of uncertain significance (VUS) in CHEK2, BUB1B, and MUTYH, which are currently considered to be of no clinical relevance. The tumor was classified as microsatellite-stable while tumor mutational burden analysis was not performed.

A follow-up FDG-PET scan performed nine months post-surgery revealed new suspicious lymphadenopathies in the left supraclavicular, mediastinal, and retroperitoneal regions, as well as metabolically active lesions in the right scapula and upper right lung lobe. Fine-needle aspiration of the left supraclavicular lymph node confirmed metastatic urothelial carcinoma (**Figure 4B**).

Metastatic disease progression was therefore established, the patient was referred to the Oncology Department and initiated first-line treatment with EV-P in January 2024. After four cycles she developed an immune-related colitis with grade 3 diarrhea. The patient also underwent a colonoscopy which revealed collagenous and apoptotic colitis consistent with immunotherapy toxicity. Pembrolizumab was therefore discontinued. The colitis completely resolved following corticosteroid treatment. EV monotherapy was continued from cycle five onward without dose reductions.

A CT scan performed after four cycles (March 26, 2024) showed stable disease per RECIST 1.1, with minimal reduction in the left supraclavicular. However, a follow-up CT scan five months after treatment initiation (June 2024, after 8 cycles of EV and 4 cycles of P) revealed multiple new suspicious millimetric pulmonary nodules. A confirmatory CT imaging performed one month later confirmed disease progression (**Figure 3**).

EV treatment was discontinued and second-line chemotherapy with carboplatin AUC 5 plus gemcitabine 1000 mg/m² was initiated in August 2024. Carboplatin was chosen because of a mildly reduced glomerular filtration rate of 45 mL/min/1.73 m2. From the third cycle onwards, both drugs were administered at a 25% reduced dosage because of emergent grade 3 thrombocytopenia and neutropenia. A CT scan after three cycles (October 31, 2024) showed a partial response according to RECIST 1.1., with both numerical and dimensional reduction of the pulmonary nodules. The case was subsequently discussed at our multidisciplinary board which, due to the good response to treatment, suggested continuing treatment with carboplatin plus gemcitabine for up to six cycles. After the completion of six chemotherapy cycles (January 2025) the patient repeated a CT staging which showed a further reduction in lung nodules (**Figure 3**). A follow-up CT scan performed in March 2025 showed no sign of disease progression.

The patient remains on active follow-up with no signs of progression, currently seven months after the last chemotherapy cycle.

## 3 Discussion and conclusions

We report two cases of patients with mUC and *BRCA 2* mutations who exhibited a short progression free survival (PFS) of 4 and 6 months to first-line EV-P therapy. In contrast they both achieved a deep and prolonged response with second-line platinum-based chemotherapy. Since international guidelines recommend the use of targeted therapies (e.g., erdafitinib in patients with mUC with FGFR alterations) as a possible second-line therapy (4, 5), at our institution, comprehensive next-generation sequencing (NGS) is routinely performed at the time of diagnosis in all patients with metastatic urothelial carcinoma, as part of standard clinical care, to determine in advance whether we can use a targeted therapy as second-line therapy. Since the results of these analyses are not always rapid, we prefer to test our patients at the diagnosis of metastatic disease in order to have these results readily available in case of disease progression. To date, between September 2021 and December 2024, a total of 23 patients were treated with EV-containing regimens at our Institute. Among these, 11 patients received EV-P as first-line treatment for mUC, starting from December 2023. Of these 11 patients, only 2 harbored pathogenic BRCA mutations, corresponding to the two cases described in our study. In the remaining 12 patients who received EV monotherapy, no BRCA alterations were identified (3).

Current guidelines recommend EV-P as the first-line standard of care for all eligible patients, regardless of tumor location or molecular profile (4, 5). However, given its recent introduction in clinical practice, predictive factors for response and survival outcomes with EV-P are largely unknown.

Our two patients had a much shorter PFS than the median reported in the EV-302 study (4 and 6 months vs 12.5 months) (3). The comparatively poor outcome on EV-P in our patients with BRCA2-mutated tumors raises questions about the potential negative predictive role of *BRCA2* mutations in mUC. In the EV-302 study, no analyses of the efficacy of EV-P based on the results of molecular analyses (e.g. pathogenic *BRCA* mutations) were reported. Therefore, to date, we have no data on the efficacy of EV-P versus platin-based therapy in patients with pathogenic *BRCA* alterations. Based on the history of our two patients one could hypothesize that patients with pathogenic *BRCA* mutation may respond better to platin-based therapy than to EV-P. And there may be some biological rationale for that.

There is a strong rationale for the observed sensitivity of BRCA-mutant mUC to platinum-based therapy. BRCA mutations are well established as predictive markers of platinum sensitivity in other malignancies (10), and further data suggest that mUC patients with BRCA mutations achieve better outcomes with first-line platinum-based chemotherapy (11) and in the perioperative setting (12).

One of the possible, yet speculative, explanations for the short response to EV-P in patients with BRCA alterations may lie on its mechanism of action. After the antibody binds to Nectin-4, the cytotoxic payload, monomethyl auristatin E (MMAE), is released. MMAE does not directly induce DNA damage; instead, it disrupts microtubule polymerization, impairing chromosomal assembly and segregation during mitosis, ultimately leading to mitotic arrest and cell death through mitotic catastrophe (13). We could hypothesize that this mechanism of action is inherently less effective in BRCA-mutated tumors, where genomic instability is a key driver of cancer progression. In such contexts, agents that cause direct DNA damage, such as platinum-based compounds, may achieve more durable disease control due to the tumor’s underlying inability to repair double-strand DNA breaks efficiently.

Our hypothesis remains speculative, as there are currently no further data to support its mechanism in patients with bladder cancer or in those receiving MMAE-based ADCs for other malignancies. In other cancer types treated with microtubule-targeting agents, the evidence is inconsistent: BRCA2-mutant tumors in some contexts exhibit limited sensitivity or inherent resistance (14–17), while in others, BRCA2 alterations appear to enhance sensitivity to these therapies (18).

Furthermore, some data suggest that alterations in DDR genes may be associated with improved outcomes in urothelial cancer patients treated with PD-1/PD-L1 inhibitors (19), complicating the interpretation of our findings. Preclinical (20) and clinical (21) data further suggest that DDR alterations could represent a biologically heterogeneous group of UCs, with individual mutations potentially linked to variable treatment sensitivities. As a result, their predictive value may be limited when assessed collectively rather than as distinct genomic entities.

When considering potential molecular predictors of response to EV-P, Nectin-4 expression assessed by immunohistochemistry was not a predictive factor of response to the EV-P combination (22). Further data on clinical and molecular predictors of EV-P response remain scarce, and genomic determinants of treatment efficacy have not yet been explored.

Predictive factors for EV monotherapy have been more extensively studied, given its earlier incorporation into clinical practice following positive outcomes in patients pretreated with platinum-based chemotherapy and immune checkpoint inhibitors in the EV-101 (23) and EV-201 (24) trials, with subsequent accelerated FDA approval in 2021 (25). For instance, the multicenter UNITE cohort analysis (26) have found that TSC1 alterations correlated with improved ORR, CDKN2A/2B alterations predicted shorter PFS, and high tumor mutational burden was associated with better OS following EV treatment. However, only 20 (11.8%) of the patients in this cohort had DDR mutations, including BRCA2, and they were not significantly associated with response to EV monotherapy or patient survival outcomes.

To sum up, while the EV-P combination represents a new, highly effective, life-prolonging standard-of-care treatment for treatment-naïve mUC patients, the identification of new clinical and genetic factors that predict treatment response, as highlighted in our report, will enable better prognostic and predictive stratification, potentially paving the way for personalized management strategies. Notably, platinum-based chemotherapy (combined with nivolumab or avelumab maintenance) remains a viable first-line treatment option for patients that are ineligible for EV-P or where it is unavailable. It can also serve as a second-line therapy after progression on EV-P in eligible patients although at the moment we have no evidence on what is the standard second line therapy in patients with urothelial carcinoma progressing after EV-P (27, 28). Both of our patients were treated with carboplatin-based chemotherapy as a second-line treatment because they were both cisplatin-unfit due to a reduced glomerular filtration rate. The positive results obtained with carboplatin-based chemotherapy as second line treatment in our two patients are encouraging, since many patients are cisplatin-unfit.

Finally, it remains uncertain whether clinical or genomic factors, such as the presence of BRCA alteration, could help identify subgroups of patients who would derive greater survival benefit from frontline platinum-based therapy even in settings where EV-P is accessible (29).Exploring this question in large prospective trials is needed. A comparative analysis of outcomes between BRCA-altered and non-HRR-altered patients treated with first and second-line platinum-based regimens could clarify whether BRCA-mutant tumors are more responsive to DNA-damaging agents or, conversely, inherently less responsive to EV-P. These two hypotheses are not mutually exclusive and warrant systematic investigation. A prospective collection of genomic and treatment response data would be an important step toward addressing this question and better defining the optimal treatment approach for patients with BRCA-mutated mUC.

Beyond the metastatic setting, the identification of predictive biomarkers for EV-P and other UC treatment regimens may have important implications in other contexts, such as the evolving perioperative landscape (30). Ongoing studies are evaluating EV-based combinations with immunotherapy in earlier disease stages (31–33), and even biomarker-driven, risk-adaptive strategies such as bladder preservation trials are being explored (34). Ultimately, the role of BRCA and other genomic alterations as predictive factors in these contexts remains a critical area for future research.

## Data Availability

The raw data supporting the conclusions of this article will be made available by the authors, without undue reservation.
