# Supplementary material for: Case Report: Could genetic factors influence the outcomes of first-line enfortumab vedotin plus pembrolizumab therapy in patients with metastatic urothelial carcinoma? Two cases of patients harbouring a BRCA mutation
Source: Front Oncol. 2025 Oct 3;15:1648230. doi: 10.3389/fonc.2025.1648230 (PMC12531046; doi:10.3389/fonc.2025.1648230)
Supplement: Supplementary file 1 [file DataSheet1.pdf]

CARE Checklist of information to include when writing a case report

CARE case report guidelines

(cc) BY-NC-ND

Topic	Item	Checklist item description	Reported on Line
Title	1	The diagnosis or intervention of primary focus followed by the words "case report"	Title
Key Words	2	2 to 5 key words that identify diagnoses or interventions in this case report, including "case report"	Keyword section
Abstract (no references)	3a	Introduction: What is unique about this case and what does it add to the scientific literature?	Abstract
	3b	Main symptoms and/or important clinical findings	Abstract
	3c	The main diagnoses, therapeutic interventions, and outcomes	Abstract
	3d	Conclusion—What is the main "take-away" lesson(s) from this case?	Abstract
Introduction	4	One or two paragraphs summarizing why this case is unique (**may include references**)	Introduction
Patient Information	5a	De-identified patient specific information.	Case One, Case two
	5b	Primary concerns and symptoms of the patient.	Case One, Case two
	5c	Medical, family, and psycho-social history including relevant genetic information	Case One, Case two
	5d	Relevant past interventions with outcomes	Case One, Case two
Clinical Findings	6	Describe significant physical examination (PE) and important clinical findings.	Case One, Case two
Timeline	7	Historical and current information from this episode of care organized as a timeline	Case One, Case two
Diagnostic Assessment	8a	Diagnostic testing (such as PE, laboratory testing, imaging, surveys).	Case One, Case two
	8b	Diagnostic challenges (such as access to testing, financial, or cultural)	N/A
	8c	Diagnosis (including other diagnoses considered)	Case One, Case two
	8d	Prognosis (such as staging in oncology) where applicable	N/A
Therapeutic Intervention	9a	Types of therapeutic intervention (such as pharmacologic, surgical, preventive, self-care)	Case One, Case two
	9b	Administration of therapeutic intervention (such as dosage, strength, duration)	Case One, Case two
	9c	Changes in therapeutic intervention (with rationale)	Case One, Case two
Follow-up and Outcomes	10a	Clinician and patient-assessed outcomes (if available)	Case One, Case two
	10b	Important follow-up diagnostic and other test results	Case One, Case two
	10c	Intervention adherence and tolerability (How was this assessed?)	Case One, Case two
	10d	Adverse and unanticipated events	Case One, Case two
Discussion	11a	A scientific discussion of the strengths AND limitations associated with this case report	Discussion and Conclusions
	11b	Discussion of the relevant medical literature **with references**.	Discussion and Conclusions
	11c	The scientific rationale for any conclusions (including assessment of possible causes)	Discussion and Conclusions
	11d	The primary "take-away" lessons of this case report (without references) in a one paragraph conclusion	Discussion and Conclusions
Patient Perspective	12	The patient should share their perspective in one to two paragraphs on the treatment(s) they received	N/A
Informed Consent	13	Did the patient give informed consent? Please provide if requested	Yes ☒ No ☐
